# Structure-activity studies of bispyridinium antinicotinics to select candidates to treat soman intoxication as part of a combined therapy

**DOI:** 10.1371/journal.pone.0318508

**Published:** 2025-02-25

**Authors:** Simon R. Turner, Christopher M. Timperley, Mike Bird, A. Christopher Green, Matthew E. Price, Helen Rice, John E. Chad, John E. H. Tattersall

**Affiliations:** 1 Chemical, Biological and Radiological Sciences Division, Defence Science and Technology Laboratory (Dstl), Porton Down, Salisbury, Wiltshire, United Kingdom; 2 School of Biological Sciences, University of Southampton, Southampton, United Kingdom; University of California Riverside School of Medicine, UNITED STATES OF AMERICA

## Abstract

The standard treatment of atropine and oximes is insufficiently effective against all organophosphorus nerve agents. Bispyridinium non-oxime nicotinic antagonists are promising components to add to treatments. One of these, MB327, improves the survival of guinea-pigs after intoxication with tabun, sarin or soman. We extend our previous study of unsubstituted bispyridinium non-oximes with C1 to C10 alkane linkers to analogues having 4-*tert-*butylpyridinium rings and the same linker range. We report their effects on nicotinic-mediated calcium responses in muscle-derived (CN21) cells where nicotinic responses were inhibited in a concentration-dependent manner. A clear structure-activity relationship resulted: the inhibitory potency increased as the linker lengthened. Previous data showed the inhibition of human acetylcholinesterase *in vitro* increased similarly and that in general the toxicity to mice increased accordingly. However, the shorter analogues MB327 (4-*tert-*butyl C3) and MB442 (unsubstituted C5) compared favourably in toxicity to some oximes used to treat nerve agent poisoning. Like MB327, the non-oxime MB442, selected by the process described, improved the survival of guinea-pigs intoxicated with soman when combined with hyoscine and physostigmine or atropine and avizafone. Our research has now afforded two compounds able to protect guinea-pigs against nerve agent toxicity through a mechanism not previously exploited deliberately for this purpose.

## Introduction

Organophosphorus (OP) nerve agents and pesticides inhibit acetylcholinesterase (AChE). This results in accumulation of acetylcholine (ACh) at muscarinic and nicotinic ACh receptors (mAChRs and nAChRs) [1–4]. Treatment focuses purely on the mAChR aspect through the use of atropine (a competitive muscarinic antagonist) [5,6]. The nicotinic effects are addressed indirectly through reactivation of the inhibited AChE with an oxime [6,7]. Each AChE-inhibitor complex differs chemically and some, after a time, resist reactivation. This means that a single oxime able to reactivate all conceivable variations of inhibited AChE is unlikely ever to be developed [8,9].

One envisages an antinicotinic component of therapy similar to atropine, which counters the muscarinic effects from AChE inhibition independent of the OP inhibitor. The difficulty is titrating sufficient competitive nicotinic neuromuscular blocker to overcome the effect of the ACh build-up and not paralyse the muscles [10–12]. This has severely hampered the pursuit of the nAChR as a therapeutic target in nerve agent poisoning with relatively few therapeutic compounds being assessed and identified as effective [13] A noncompetitive antagonist, whose effect is not annulled by increasing ACh, is appealing. Some bispyridiniums, including oximes [14,15], ameliorate OP poisoning via this action, in line with their ability to block the nAChR open ion channel [16]. Such non-competitive block is promising as antagonism increases as channel activation increases. This is an opposite result to that from a competitive antagonist and can mitigate the effects of overstimulation of nAChRs.

**Fig 1:**
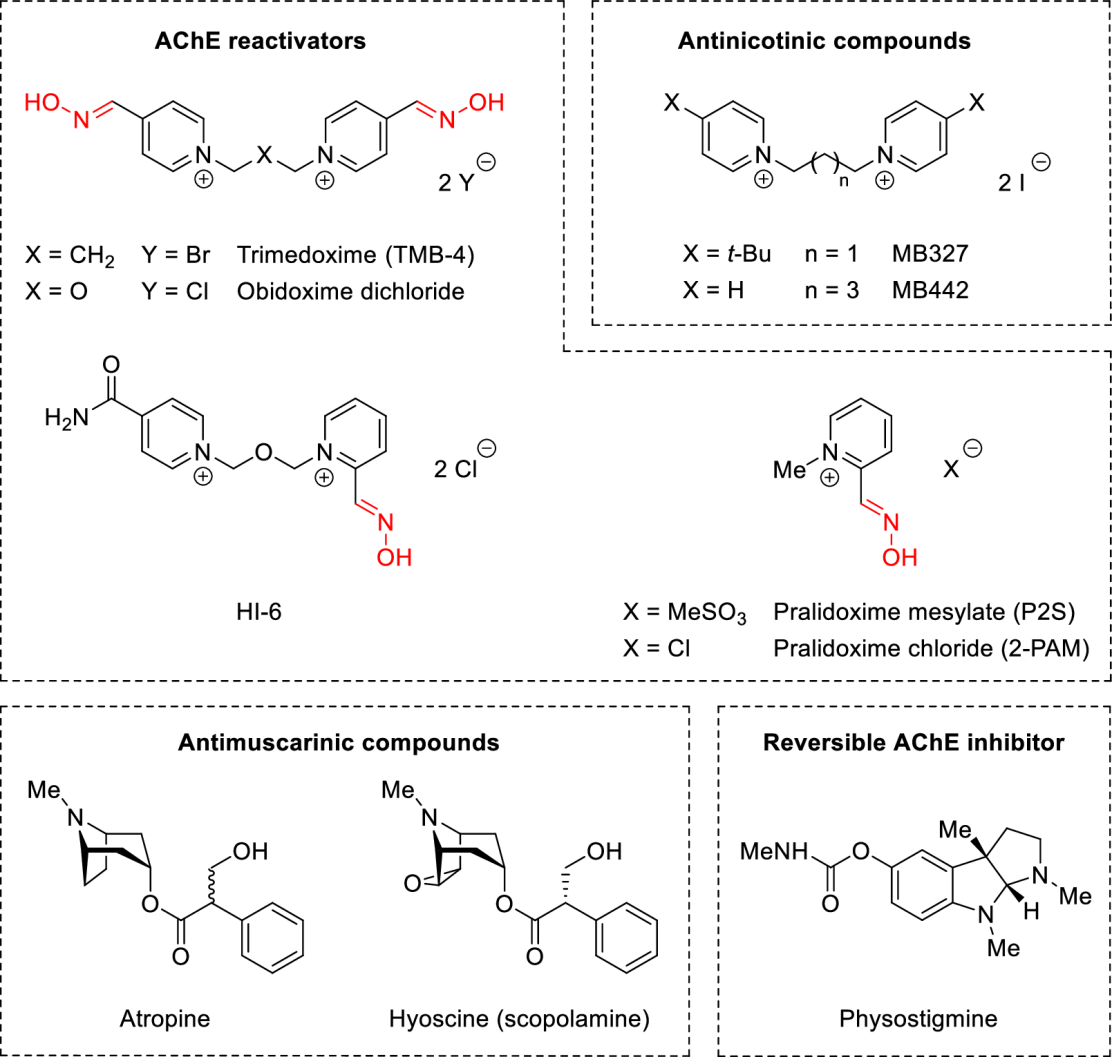
AChE reactivators, antinicotinics, antimuscarinics and the reversible AChE inhibitor discussed here, connected with nerve agent medical countermeasures. Reactivators (top left) contain an oxime group (coloured red) and the antinicotinics (top right) do not. The reactivators are therefore termed ‘oximes’ and the antinicotinics ‘non-oximes’; the non-oximes cannot reactivate nerve-agent inhibited AChE.

Building on hints that such compounds were effective in the treatment of soman-intoxicated mice for reasons not understood [17–20], we synthesised the 4-*tert*-butyl bispyridinium non-oxime MB327 (Fig 1), which blocks the open ion channel of the muscle-type nAChR [21,22]. This non-competitive block, in addition to countering the neuromuscular block from soman *in vitro*, and when combined with other drugs, gave protection to animals exposed to nerve agents [9]. Certain unsubstituted bispyridinium compounds interacted with muscle and neuronal nAChRs, and the strength of the interaction depended on the length of the alkane chain linking the pyridinium rings [23].

Here, we show that a series of 4-*tert*-butyl substituted compounds including MB327 have a similar structure-activity relationship and we characterise the ability of both sets of compounds to recover neuromuscular function in guinea-pig diaphragm *in vitro* following intoxication by soman. We also prove that the most effective unsubstituted compound in this assay, MB442 (Fig 1), protects guinea-pigs from soman *in vivo* like MB327 when administered with hyoscine and physostigmine or atropine and avizafone.

## Materials and methods

### Drugs and chemicals

Bis(*tert*-butylpyridinium) diiodides were made by treating 4-*tert*-butylpyridine with α,ω-diiodoalkanes (2:1 molar ratio), analogous to reported methods [9,24,25]. They were > 98% pure and soman was 98% pure by ^1^H NMR spectroscopy. Other chemicals were purchased from Sigma-Aldrich (Gillingham, Dorset, UK) unless stated otherwise.

### Cell culture

CN21 cells were derived from the TE671 human rhabdomyosarcoma cell line with the ε-subunit stably transfected such that these cells express both the foetal (α1, γ, α1, ß1, δ) and the adult human (α1, ε, α1, ß1, δ) forms of the muscle nicotinic receptor. CN21 cells were a kind gift from Dr. David Beeson (Institute of Molecular Medicine, John Radcliffe Hospital, Oxford, UK) [26]. The cells were grown using standard culture techniques in Dulbecco’s Modified Eagle’s Medium (Sigma-Aldrich, UK) with 10% Foetal Bovine Serum (Invitrogen, UK), 50 units·ml^−1^ penicillin, 50 µg·ml^−1^ streptomycin, 2 mM L-glutamine (Sigma-Aldrich, UK) and 0.5 mg·ml^−1^ geneticin (Invitrogen, UK) in 150 cm^2^ cell culture flasks until ~70–80% confluent (in a humidified atmosphere in an incubator at 36.5 °C with 5% CO_2_). Cells were harvested using 0.25% trypsin/ethylenediamine tetraacetic acid (EDTA) solution (Sigma-Aldrich, UK) in Ca^2+^ /Mg^2+^ -free phosphate buffered saline, and collected by centrifugation (100 × g_max_, 4 min). Cells were replated into culture flasks at split ratios of 1:6 to 1:10, and used in experiments between passages 2 and 8 after recovery from cryopreservation.

### Nicotinic calcium response assay

CN21 cells were plated onto clear-bottomed, black-walled, tissue culture-treated 96-well plates (Corning Costar^®^) at ~20,000 cells per well in 100 µl of medium (so they approached confluence after 24 h). The growth medium was removed, leaving the cells adhering to the bottom of the plate and Calcium 4 assay kit dye (50 µl; Fluo-4 acetoxymethylester, FLIPR Calcium 4 assay kit, Molecular Devices, California, USA) in a HEPES-buffered balanced salt solution (NaCl 135 mM, KCl 5.4 mM, CaCl_2_ 1 mM, MgCl_2_ 1 mM, HEPES acid 5 mM, NaHCO_3_ 3.6 mM, D-glucose 10 mM, pH 7.4 with NaOH) was added. The cells were incubated in the dark for ~30 min. They were not washed prior to the assay, as the FLIPR Calcium 4 assay kit contained a quenching dye to minimise fluorescence from extracellular de-esterified (fluorescent) Fluo-4. The dye loading solution contained atropine (20 µM) to block the muscarinic response and pharmacologically isolate the nicotinic response to ACh.

Test compounds were dissolved in HEPES-buffered solution on the day of the experiment. After the cells were incubated with the dye, dilutions of this stock solution (12 µl) were added to each well on the cell plate to give the final desired concentrations once the agonist was added. The first and last columns received a vehicle control to compare the compound response to the vehicle at the start and end of each experiment, and to evaluate time-dependent effects. Each compound was tested at 10 different concentrations in duplicate on each plate and each experiment was conducted 3 times.

The plates were placed in a FlexStation II fluorescence plate reader (Molecular Devices, UK). Fluorescence intensity was measured at ~1 s intervals prior to and after adding 20 µM ACh (58 µl) to give a final concentration per well of ~10 µM, which yielded ~80% of the maximal response (EC_80_). Excitation and emission wavelengths were 485 nm and 525 nm respectively with a 515 nm cut-off. Measurements were made at room temperature (~21 °C). Agonist additions were via the plate reader’s auto-dispenser. Baseline fluorescence was recorded for 20 s and ACh was added. Further measurements of fluorescence for 80 s were taken to track the agonist-induced response.

Responses were quantified as the maximum response expressed as a percentage of the average baseline values. The data were normalised to the mean vehicle control values for wells in the corresponding row. Responses were fitted to a standard four-parameter logistic equation using GraphPad Prism v. 4.00 (GraphPad Software, California, USA). A log half-maximal inhibitory concentration (log IC_50_) and Hill slope with standard errors of the mean (SEM) were determined. For analysis of the lower potency compounds, curve fitting assumed a maximal inhibition equivalent to that obtained with the higher potency C8–C10 compounds where maximal inhibition could be fitted. This value was constrained in analyses of the lower potency data, allowing estimation of the IC_50_.

### Diaphragm muscle assay

Male Dunkin-Hartley guinea pigs weighing between 245 g and 642 g (mean = 344 g) were euthanized in accordance with Schedule 1 of the UK Animals (Scientific Procedures) Act 1986, by concussion of the brain followed by exsanguination, and left and right hemidiaphragms were prepared [27]. Preparations were suspended in a modified Tyrode solution containing (in mM): NaCl 137, NaHCO_3_ 12, NaH_2_PO_4_ 1, KCl 5, MgCl_2_ 1, CaCl_2_ 2 and glucose 25 at ∼36.5 °C and gassed with 95% O_2_/5% CO_2_, pH 7.4. They were stimulated via the phrenic nerve through bipolar platinum electrodes with 0.25 ms rectangular pulses. The stimulus intensity was adjusted for supramaximal stimulation of the preparations. Muscle contractions were recorded from a baseline tension of 4 g using a Biopac TSD125D isometric force transducer (Biopac Systems Inc., California, USA) and digitised at 500 Hz using eDacq v. 1.1.17 (Electro-Medical Measurement Systems, Hampshire, UK).

Single twitch responses every 10 s were recorded. Every 15 min, a 50 Hz tetanus was elicited for 3 s. This pattern approximated well the average frequency of phrenic nerve impulses during normal inspiration in animals [28]. Preparations were allowed to equilibrate for at least 45 min after the start of stimulation. Following control tetani, soman was added to the tissue bath to 100 nM final concentration. Soman was used because the rapid ageing of inhibited AChE eliminated any possibility of reactivation [16]. The preparation was exposed to soman for 30 min. The excess of soman was washed out. The preparation was left for 30 min to age the inhibited AChE before adding the test compound. Each compound was studied for its effects on the preparation at 10, 30 and 100 µM. Compounds were made as 10 mM stocks in water and the appropriate volume was added to the tissue bath to give the final concentration.

Tissues were exposed to each concentration for 30 min and were applied cumulatively to the tissue bath. After applying the highest concentration, the compound was washed off and a tetanus test was recorded 30 min after washing. Each experiment was repeated on separate hemidiaphragms from 4 different animals (n = 4) and the results compared with time-matched vehicle controls from the contralateral side of the same animals.

Neuromuscular function was assessed by measuring the area under the curve of the response to the tetanic stimulation, which indicated the maximum tension the diaphragm could produce and how it maintained across the 3 s of the tetanus test. The area under the response was expressed as a percentage of the tetanus recorded immediately prior to applying the soman. Differences of the vehicle- and drug-treated groups with each concentration of test compound were compared by a 2-way analysis of variance (ANOVA) followed by a Bonferroni post hoc test to detect any statistically significant difference (at p < 0.05) between the groups and the concentration those differences occurred at (if at all) (GraphPad Prism v. 4.00 for Windows, GraphPad).

## In vivo protection

All work with living animals involving regulated procedures at Dstl is conducted in accordance with the UK Animals (Scientific Procedures) Act 1986 (ASPA). Dstl is a Licenced Establishment under the ASPA, and all work involving regulated procedures undergoes review and approval by Dstl’s Animal Welfare and Ethical Review Body (AWERB) before an application is made to the UK Home Office for a Project Licence that independently authorises the work to be undertaken. All regulated procedures are performed by suitably trained and qualified individuals holding Personal Project Licences (PPLs) in accordance with ASPA. The UK Home Office licence approving the work in this article was Project Licence 30/02864, “Medical countermeasures to chemical agents”. Experiments described in Fig 6 were carried out under PPLs 30/1835 and 30/2367, both entitled “Anticholinesterases and medical countermeasures”.

**Fig 2:**
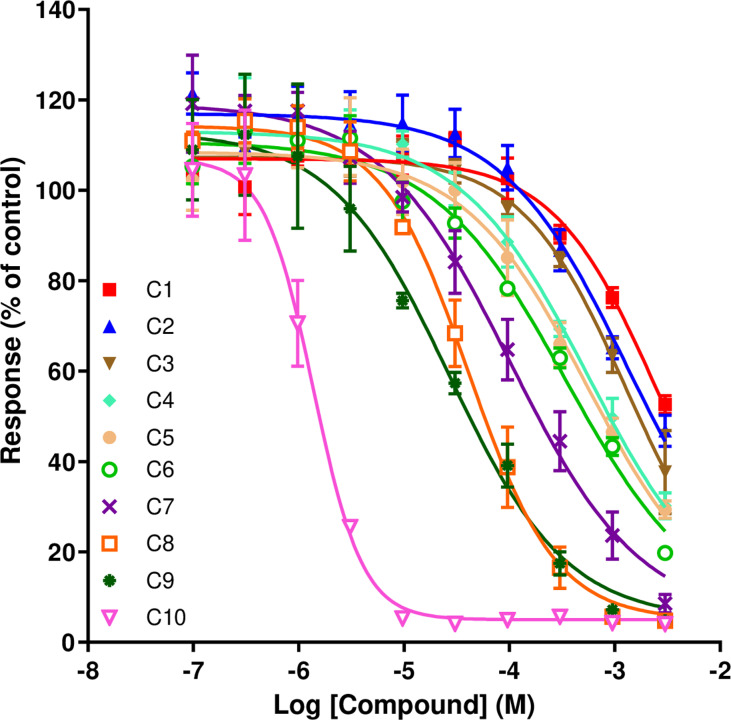
Influence of linker length of the 4-*tert*-butyl compounds on the effect of the Ca^2+^ response to ACh (10 µM) measured in CN21 cells. Data are the mean ± SEM from 3 experiments carried out in duplicate on each plate. A standard four-parameter logistic equation was used to fit the curves.

**Fig 3:**
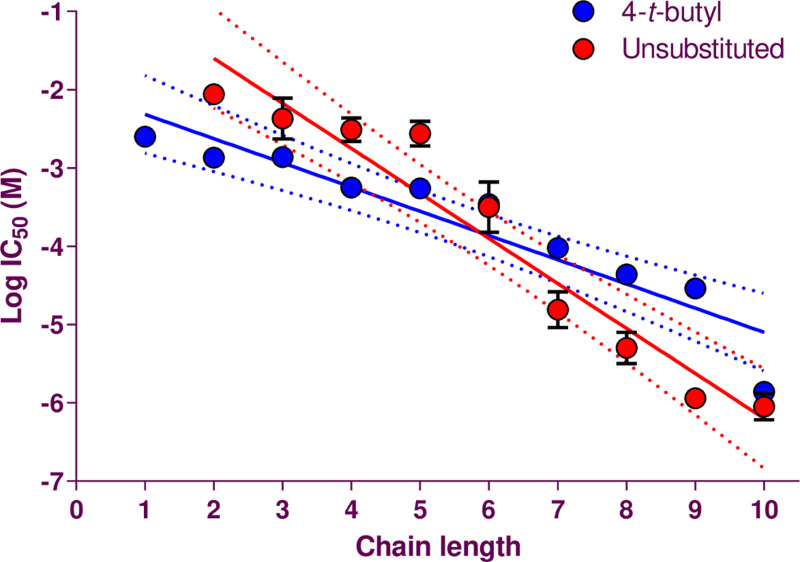
Relationship of linker length of the bispyridinium compounds and the log IC_50_ in CN21 cells. The solid lines indicate the linear regression fit of the data: 4-*tert*-butyl, **y** = −0.309x – 2.007, r^2^ = 0.880; unsubstituted, −0.575x − 0.450, r^2^ = 0.936. The broken lines show the 95% confidence intervals of the fit.

**Fig 4:**
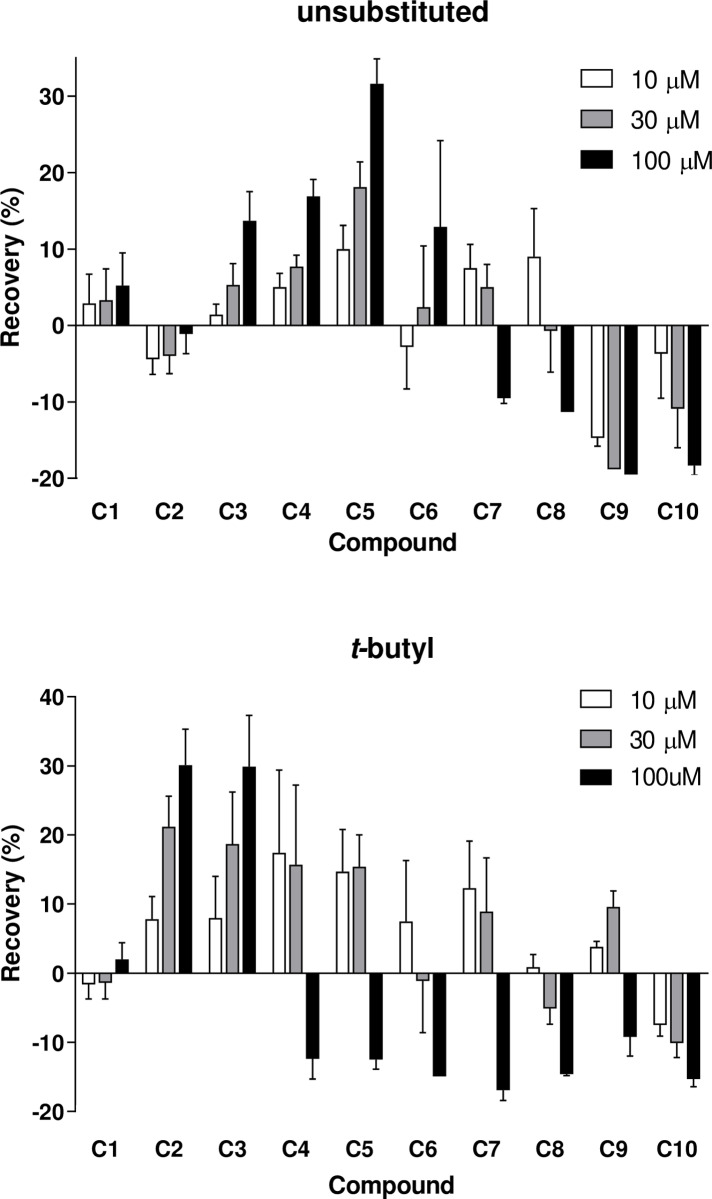
Effect of linker length of the unsubstituted (top) and 4-*tert*-butyl (bottom) bispyridinium compounds on the soman-poisoned guinea pig diaphragm. Data corrected for vehicle and time effects; they are the mean ± SEM of four preparations (n = 4).

**Fig 5:**
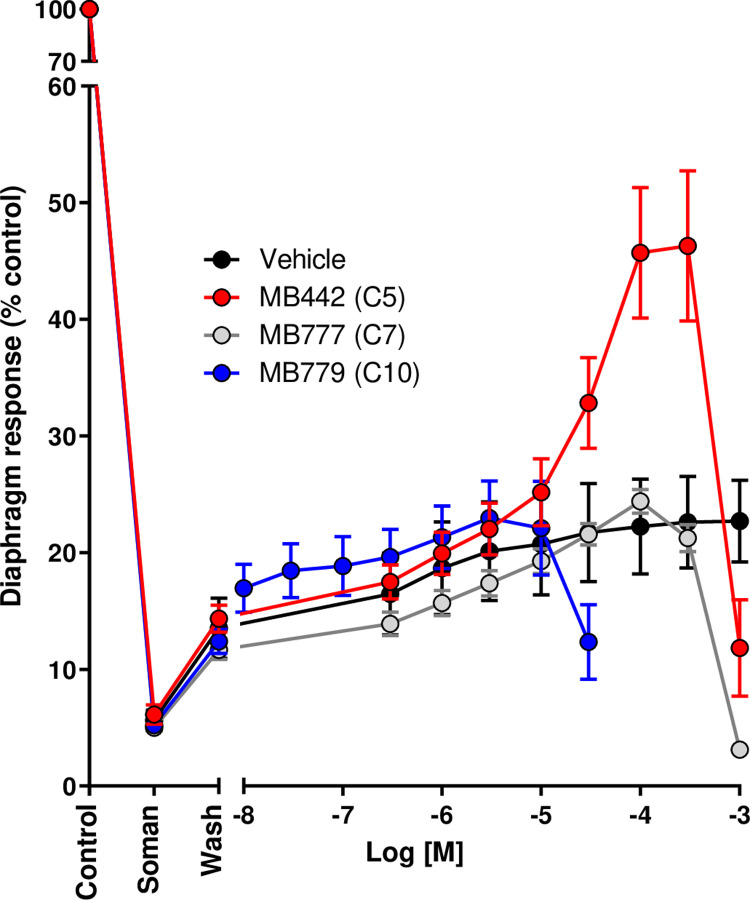
Effect of the unsubstituted compounds C5, C7 and C10 over a concentration range that produced no effect, possible recovery and inhibition of diaphragm response. The points are percentages of the value of the pretreatment control. The data are means ± SEM of 4 preparations. The time-matched vehicle control group is also shown.

**Fig 6:**
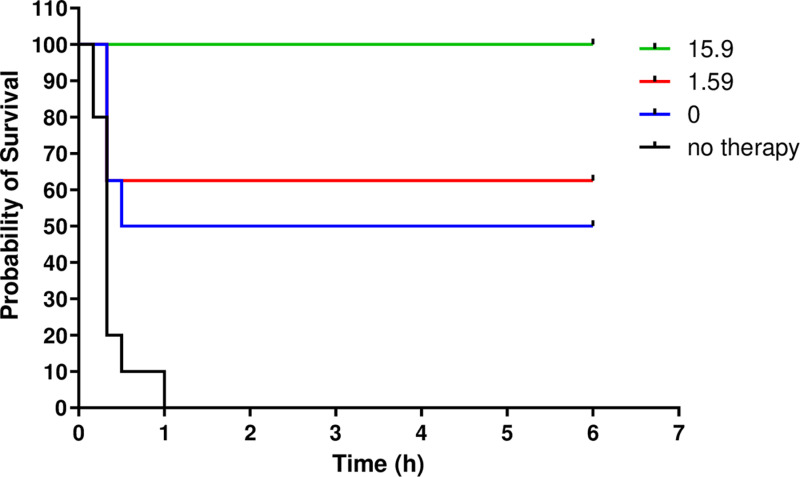
Protection by MB442 versus soman intoxication: 6 h survival curves for guinea-pigs treated with 5 × LD_50_ soman and 1 min afterwards with a mixture of physostigmine (0.2 mg·kg^−1^), hyoscine (4 mg·kg^−1^) and two doses of MB442 (1.59 or 15.9 mg·kg^−1^). The control animals were administered saline vehicle alone after the soman. Treatment groups contained 8 animals. The saline control group comprised 10 animals.

The effectiveness of MB442 (unsubstituted C5 bispyridinium diiodide) combined with hyoscine and physostigmine or with atropine and avizafone was determined against soman intoxication. Male Dunkin-Hartley guinea-pigs were used for this study: they are an accepted model for nerve agent intoxication and treatment, and previous experience was used to enable this assessment of MB442 [29,30]. For these studies, the guinea-pigs were injected subcutaneously in the interscapular region with soman solutions, and 1 min later each was injected intramuscularly in the upper hind limb with the therapeutic mixtures, as now described.

### Protection against a fixed agent dose challenge

Male Dunkin-Hartley guinea pigs, 292 g to 396 g (mean 339.4 ± 23.1 standard deviation (S.D.); n = 34) were injected with approximately 5 × LD_50_ soman (135 μg·kg^−1^), followed by a mixture of physostigmine salicylate (0.2 mg·kg^−1^), hyoscine hydrobromide (4 mg·kg^−1^) and no further therapy, or MB442 at either of two doses (1.59 or 15.9 mg·kg^−1^). Groups of eight animals were used for the two combinations of therapy and animals were randomly assigned to these groups. Group size was based on previous experience [21,29,30], with this experimental design being able to demonstrate statistically significant benefits of related antinicotinic compounds above that provided by the physostigmine and hyoscine alone [21]. Following challenge, the guinea pigs were checked for signs of intoxication every 15 min for 4 h after soman administration, and henceforth hourly up to 6 h. Times to death were recorded. Administrations to different experimental groups were randomised in time (up to 6 animals were challenged on each experimental day). Staff were not blinded to the experimental conditions due to the hazardous nature of these studies. Control animals (no therapy, n = 10) received a saline vehicle after the soman. Data were plotted as Kaplan-Meier survival curves and compared using a log-rank (Mantel-Cox) test in GraphPad Prism v. 8.0.1.

### Protection ratio studies

Separately, protection was assessed by deriving 24-h LD_50_ values for soman in the presence of different therapies [31]. Male Dunkin-Hartley guinea-pigs weighing between 273 g and 348 g (mean 319.1 ± 22.4 S.D.; n = 30) were injected with soman subcutaneously (range: 14.5–114.8 µg·kg^−1^) followed by a therapy of atropine (3 mg·kg^−1^) and avizafone (3 mg·kg^−1^), and either no additional therapy, or MB442 at either of two doses (9.62 or 28.78 mg·kg^−1^). The LD_50_ values were derived using an adaptive probit method [31]. Due to the adaptive design of this study and for safety reasons due to the hazardous nature of the challenge agent, staff were not blinded to the experimental conditions.

## Results

### CN21 cells

A Ca^2+^ influx could still be measured using Fluo-4 (Ca^2+^ -sensitive fluorescent dye), when CN21 cells were exposed to ACh and atropine (20 µM) to stop the muscarinic response. This response reached a maximum in < 10 s. It was blocked completely by adding the nicotinic antagonist d-tubocurarine (50 µM) [21]. No response resulted when KCl (10–100 mM) was employed to depolarise the cells in the absence of adding ACh. This confirmed that any response was due to activation of nAChRs [23]. An agonist concentration of 10 µM ACh (~EC_80_) of the response was selected for further experiments with the bispyridinium chemicals.

### Effect of test compounds on CN21 cells

Ten concentrations of the 4-*tert*-butyl substituted compounds decreasing in half-log steps from 3 mM were tested on the CN21 cells. A concentration-dependent inhibition of the nicotinic response was observed with the potency increasing with linker length (Fig 2). Maximum potency occurred with the C10 compound (MB786). It had similar potency to the unsubstituted C9 (MB778) and C10 (MB779) compounds [23]. In the *tert*-butyl series, potency rose steadily as the linker extended from C1 to C9 (Table 1). A large increase from the C9 (MB582) to C10 (MB 786) was observed. Only the 4-*tert*-butyl C10 produced a Hill slope near −2; the other compounds gave slopes closer to −1.



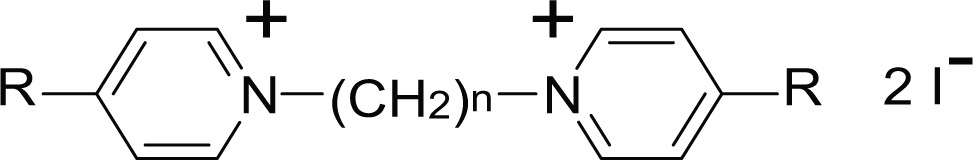



**Table 1:** Log IC_50_ values for inhibition of nicotinic responses in CN21 cells by the substances.

	Tert-butyl series: R = C(CH_3_)_3_			Unsubstituted series: R = H ^a^		
n	Test compound	Log IC_50_ [M]	Hill slope	Test compound	Log IC_50_ [M]	Hill slope
1	MB780	−2.60 ± 0.04	−0.88 ± 0.01	MB775	DNC	DNC
2	MB583	−2.87 ± 0.06	−0.74 ± 0.08	MB520	−2.06 ± 0.04	−0.75 ± 0.06
3	MB327	−2.86 ± 0.16	−0.89 ± 0.10	MB408	−2.37 ± 0. 26	−0.73 ± 0.06
4	MB781	−3.25 ± 0.09	−0.72 ± 0.01	MB444	−2.51 ± 0.15	−0.72 ± 0.05
5	MB782	−3.26 ± 0.05	−0.74 ± 0.11	MB442	−2.56 ± 0.16	−0.60 ± 0.13
6	MB783	−3.46 ± 0.04	−0.69 ± 0.03	MB776	−3.50 ± 0.32	−0.50 ± 0.02
7	MB784	−4.02 ± 0.28	−0.73 ± 0.07	MB777	−4.81 ± 0.23	−0.72 ± 0.01
8	MB785	−4.36 ± 0.14	−1.15 ± 0.12	MB505	−5.30 ± 0.20	−0.99 ± 0.07
9	MB582	−4.54 ± 0.11	−0.81 ± 0.04	MB778	−5.94 ± 0.11	−1.56 ± 0.11
10	MB786	−5.86 ± 0.06	−1.93 ± 0.21	MB779	−6.05 ± 0.17	−1.34 ± 0.14

Structures of the compounds appear above. Data are mean ± standard error of mean (SEM) from the best fit of the Hill equation. a. [23]. DNC = data did not converge: not possible to obtain log IC_50_ or Hill slope.

Fig 3 shows IC_50_ values and linker lengths of 4-*tert*-butyl analogues compared to those of the unsubstituted ones [23]. Both series inhibited the nicotinic response more potently the longer the linker. The 4-*tert*-butyl compounds were generally more potent than unsubstituted counterparts up to the C5 linker. For longer linkers, the unsubstituted compounds were more potent.

### Effect of test compounds on acetylcholinesterase

A desired drug with antinicotinic action should not be a potent AChE inhibitor because this imparts toxicity. Human acetylcholinesterase (hAChE) inhibition and toxicity data for the drugs tested appear in Table 2 [23]. Both series increased in potency as the linker lengthened. The 4-*tert*-butyl C1 to C3 analogues weakly affected activity (IC_50_ values in the hundreds of µM). The C4 to C10 compounds inhibited hAChE with increased potency (the IC_50_ values for the C6 to C10 analogues were < 1 µM). The potencies of the 4-*tert*-butyl compounds exceeded those of the unsubstituted series.



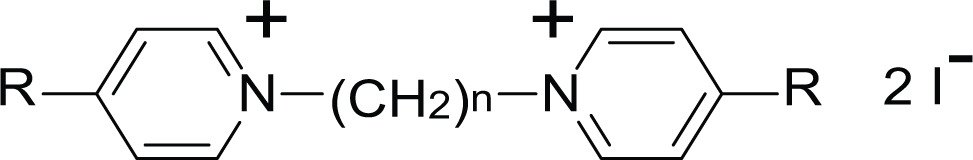



**Table 2:** IC_50_ values for hAChE inactivation and intravenous (i.v.) and intramuscular (i.m.) LD_50_ values in mice for the substances and some oximes used to treat nerve agent intoxication.

Compounds	hAChE IC_50_ (μM)^a^				LD_50_ i.v. mice (i.m. when number is in brackets) (mg·kg^−1^)	
**Bispyridiniums**						
Chain-length n	MB code	R = *t-*Bu ^a^	MB code	R = H ^a^	R = *t-*Bu	R = H ^a,b^
C1	MB780	974 ± 0.0	MB775	1218 ± 0.0	NT	NT
C2	MB583	555 ± 240	MB520	6981 ± 0.0	NT	225
C3	MB327	404 ± 176	MB408	3700 ± 0.0	(167) ^c^	180 (603)
C4	MB781	35 ± 26	MB444	1420 ± 40	NT	55 (203)
C5	MB782	4 ± 0.8	MB442	711 ± 239	NT	32 (116)
C6	MB783	0.6 ± 0.2	MB776	84 ± 4	NT	26
C7	MB784	0.6 ± 0.2	MB777	16 ± 2	NT	NT
C8	MB785	0.3 ± 0.0	MB505	1.4 ± 0.7	(33) ^d^	NT
C9	MB582	0.2 ± 0.1	MB778	0.3 ± 0.0	NT	NT
C10	MB786	0.1 ± 0.0	MB779	0.2 ± 0.0	NT	15 ^e^ (64)
**Common oximes**						
HI-6 dichloride		NT		NT	24 ± 4	178 (671)
P2S		NT		NT	NT	116
2-PAM chloride		NT		NT	210 ± 14	93 (180) ^f^
Obidoxime		NT		NT	280 ± 28	70 (188)

NT = Not tested. a. [32]. b. [33]. c. [34]. d. [35]. e. The C10 dibromide was tested in mice in a rotating drum. The i.v. ED_50_ was 1.9 mg·kg^−1^ and i.v. LD_50_ was 6.5 mg·kg^−1^ [36]. Paralysis was curariform. The C10 dinitrate is a neuromuscular blocking agent [37]. It had ‘considerable anticholinesterase activity’ and ‘at the same time exhibits a direct action causing depolarisation of the motor end plate. To produce a depolarising block comparable with that resulting from decamethonium a dose of about 200 times greater is required’ [37]. LD_50_ i.v. in mice was 7.2 mg·kg^−1^. f. Baxter Healthcare (Newbury, UK).

### Recovery in soman-inhibited diaphragm preparations

We studied the ability of the compounds to improve neuromuscular function post-soman administration in the guinea-pig phrenic nerve-hemidiaphragm preparation after aging of the inhibited AChE (to discount reactivation in any recovery of function). For unsubstituted bispyridiniums, recovery increased with length to C5 (MB442) and then declined (Fig 4). Up to C6, recovery improved with concentration, to the maximum (100 µM). For C7 and C8, the recovery was greatest at low concentration (10 µM). The C9 and C10 compounds did not produce recovery at the three concentrations tested and abolished responses in single twitch and tetanus tests. When a wash was performed, those preparations treated with C9 and C10 that abolished the response did not recover completely. Afterwards we observed a slow recovery. All the other responses for the compounds returned to a state similar to the vehicle-treated group after the wash.

For the 4-*tert*-butyl series, maximum recovery occurred for C2 (MB583) and C3 (MB327) (Fig 4). C4 to C10 abolished twitch and tetanus responses at the highest concentration. Recovery at the low and medium concentrations occurred for C4, C5, C7 and C9 (and for C6 at the low concentration only). Upon washing, all responses reversed similar to the vehicle-treated group, except in preparations exposed to C10. These recovered slowly, similar to those for the unsubstituted C9 and C10 compounds. The responses failed to recover to the level of the time-matched vehicle control. Overall, the results for the two series resembled those recorded in rat diaphragms [32].

To examine the effect of selected compounds over a broader concentration range, a concentration-response curve for the unsubstituted compounds C5 (MB442), C7 (MB777) and C10 (MB779) was produced. The C5 analogue gave a good recovery of diaphragm response. Extending the linker inhibited the response over the same concentration range. Analysis of the effects of these compounds on the nicotinic response in CN21 cells had showed the C7 and C10 to inhibit more potently the nicotinic response than the C5 compound. These experiments enabled assessment of how nicotinic antagonism contributed to inhibition of diaphragm response and recovery of its function following soman intoxication. Fig 5 shows the effects of the compounds and a vehicle control group. The C5 compound produced concentration-dependent recovery of the tetanic response up to 100 µM. Concentrations over 300 µM inhibited the response. Neither the C7 nor C10 materials produced any recovery above the vehicle group. The C7 inhibited the response less potently than the C10 analogue.

### Protection versus soman intoxication in vivo

The action of the most effective unsubstituted compound C5 (MB442) added to hyoscine and physostigmine was assessed as a mixture versus soman intoxication. The rationale for this triple therapy appears elsewhere [9,29,30]. Briefly, hyoscine (Fig 1), an antimuscarinic acting like atropine, antagonises the effect of the excess ACh at the mAChRs. Physostigmine (Fig 1), a reversible AChE blocker, inhibits some of the AChE transiently, which protects it from irreversible inhibition by the nerve agent.

Earlier, a mixture of MB327 dimethanesulfonate, hyoscine and physostigmine had enabled guinea-pigs to survive challenges of the nerve agents sarin, soman or tabun [9]. Protection against these agents was similar to that versus soman here. Guinea pigs were challenged with 5 × LD_50_ of soman s.c. and were treated i.m. a minute later with hyoscine and physostigmine, or these combined with MB442. Saline without therapy drugs was injected into control animals. The survival information is presented in Fig 6.

All of the control group animals (n = 10) died in the first hour after soman exposure. Four of those given therapy containing only hyoscine and physostigmine died in the first hour. The other four guinea-pigs survived until 6 h after receiving the soman. The survival curves differed significantly (log-rank test, p < 0.011). A dose-dependent increase in survival was evident when MB442 was added to the therapy. Five out of 8 guinea-pigs survived to 6 h when it contained 1.59 mg·kg^−1^ (p = 0.0039) in comparison to the controls, and all guinea-pigs survived when it contained 15.9 mg·kg^−1^ (p < 0.0001) of MB442. The survival with the highest dose of MB442 differed significantly from that for the therapy containing only hyoscine and physostigmine (p = 0.0247).

Protection against soman intoxication by MB442 in the presence of atropine and avizafone—a water-soluble prodrug of the anticonvulsant diazepam [6,9]—was assessed by the change in the 24-h LD_50_ of soman. The LD_50_ of soman increased in a dose-dependent manner with increasing MB442 (Table 3). The protection ratio was greatest at the highest MB442 dose (28.78 mg·kg^−1^), but adverse effects—including apnoea, dyspnoea and flaccid paralysis—were observed in 1 out of 3 guinea-pigs injected at this dose in the absence of a soman challenge.

**Table 3:** Increase in 24-h LD_50_ (±SE of the estimate) of soman afforded by MB442 at two doses after administration in combination with atropine sulfate and avizafone (3 mg·kg^−1^ each).

Dose of MB442 (mg·kg^−1^)^a^	Soman LD_50_ (µg·kg^−1^)	Protection ratio^b^
No therapy	29.3 ± 0.9	–
Therapy control (atropine/avizafone only)	33.52 ± 4.1	1.14 ± 0.14
9.62	41.67 ± 7.54	1.42 ± 0.26
28.78	50.87 ± 11.47	1.74 ± 0.40

^a^MB442 with atropine sulfate (3 mg·kg^−1^) and avizafone (3 mg·kg^−1^) was administered at the doses shown 1 min after a s.c. challenge with soman. Data for soman alone from our previous work (Whitfield et al. [unpublished]).

^b^Protection ratios calculated compared to the “no therapy” control group.

## Discussion

Bispyridiniums such as MB442 and MB327 provide effective adjuncts to anti-muscarinic treatment, restoring function in nerve agent-exposed neuromuscular preparations *in vitro* and promoting survival *in vivo* over and above that seen with anti-muscarinic and oxime-based therapies. They have no oxime group and therefore cannot reactivate nerve-agent inhibited AChE. The binding of the unsubstituted bispyridinium compounds to mAChRs is low compared to atropine [20,23,38]. It is unlikely the compounds contribute an appreciable antimuscarinic effect compared to atropine or hyoscine in any nerve agent therapy. However, even their modest antimuscarinic action may contribute to the antidotal effect. The bispyridiniums do however exhibit a favourable antinicotinic action [39] and it is action at this target that is believed to underpin their therapeutic mechanism.

This study demonstrates bispyridinium compounds inhibit nAChR-mediated calcium responses in CN21 cells and that the potency of this inhibition increases as the linker between the pyridinium rings lengthens. Compounds with shorter linkers produced recovery of tetanic contraction in soman-inhibited diaphragm muscle tissue, while those with longer linkers inhibited contraction. Compounds that produced maximum recovery in diaphragm preparations also provided protection against soman poisoning *in vivo* when administered in therapeutic drug combinations.

The studies presented here provide structure activity data that focuses predominantly on the linker length in two series of bispyridinium compounds. Whilst other structural features have been examined [40], linker length was the most directly linked to activity at the nAChR target. Altering the linker length profoundly influenced the activities of the unsubstituted and the 4-*tert*-butyl analogues. This confirms that the molecular length and shape of such bispyridinium compounds influence strongly the degree of nicotinic antagonism. The relationship between linker length and potency was more marked for the unsubstituted series than the 4-*tert*-butyl compounds (slopes of −0.309 ± 0.0403 and −0.575 ± 0.0567, respectively; Fig 3). The 4-*tert*-butyl compounds were more potent than their unsubstituted counterparts up to the length of C5, beyond which the unsubstituted compounds became more potent. For the unsubstituted series, the slope of the relationship between the C5 and C6 drugs changed [23], which has also been found for polymethylene bis-trimethylammonium compounds [41]. This was unapparent for the 4-*tert*-butyl compounds, although a > 1 log unit change in IC_50_ between the C9 and C10 members was observed, accompanied by a change in Hill slope from −0.81 to −1.93. All the other compounds gave much lower Hill slopes, around −1 or less.

Interpretation of the Hill slopes is complicated by the mixed populations of nicotinic receptors in CN21 cells expressing foetal (α1, γ, α1, ß1, δ) and adult (α1, ε, α1, ß1, δ) human muscle nAChRs [21,26]. Variations in Hill coefficients might reflect different degrees of cooperativity of the interactions of the bispyridinium compounds with these receptor subtypes. Although CN21 cells do not appear to express voltage-gated Ca^2 +^ channels [23,42,43], the presence of a Ca^2 +^ -mediated Ca^2 +^ release could also result in non-linearity that could manifest in differences in the Hill coefficient.

Concentration-dependent recovery of soman-poisoned diaphragm was observed with the C3 to C6 unsubstituted compounds (peaking at C5), and C2 and C3 compounds in the 4-*tert*-butyl series (Fig 4). In the former series, C7 and C8 produced recovery at lower concentrations, but this changed to inhibition of the response at higher concentrations. A similar pattern emerged in the 4-*tert*-butyl series: C5, C6, C7 and C9 produced recovery at lower concentrations, but were inhibitory at higher concentrations.

The fuller concentration-response data for the C5, C7 and C10 unsubstituted analogues confirmed the lack of recovery from these longer compounds was not because their concentrations were too high. Recovery from the unsubstituted C5 analogue (MB442) was greatest at 100 and 300 µM and declined greatly at 1 mM. In contrast, in this series, neither the C7 (MB777) or C10 (MB779) analogues produced any recovery above that of the vehicle control group. The C7 compound inhibited the response more potently than the C10. These results, and the effects of the compounds on nicotinic responses in CN21 cells, suggest inhibition of diaphragm function is due to antagonism of the nicotinic response to the extent of transmission failure. The C5 recovered diaphragm function, whereas the C7 and C10 analogues were unable to, even over a broad concentration range. If recovery and inhibition solely result from nicotinic antagonism, it appears the nature of this antagonism differs between the C5 and C7/C10 analogues.

Compounds that inhibited the nicotinic response in CN21 cells could competitively or non-competitively antagonise nAChRs, or comprise agonists partially activating or desensitising them. Compounds that compete with ACh for the receptor or block its channel in the closed state, or desensitise the receptor, may be more effective at inhibiting the response in this assay than fast open-channel blockers. Antagonists that completely block the current flow at the receptors they bind to could be more effective at inhibiting the response than fast open channel-blockers, which modulate but do not completely abolish current flow through the ion channel. Because of the kinetics of the response and blocking compounds, it was impossible to attribute competitive or non-competitive mechanisms of action to these chemicals in the fluorescence assay [23].

Discovery of the precise mechanism underlying the block will necessitate more experiments, e.g., via a single channel assay [21]. The concentration-response curves for the weaker antagonists generally had shallower Hill slopes than those for the more potent blockers. Drugs with shallow Hill slopes could be more attractive as medical therapies against the nicotinic effects from nerve agent intoxication. They could have a larger therapeutic window before the drug itself becomes toxic through an overwhelming block of the nAChR function. Drugs that have a steep concentration-response curve may need a more cautious titration of the therapeutic dose. This is to ensure amelioration of over-stimulation of nAChRs before their function is lost through increasing antagonist concentrations. The diaphragm results support this: the most effective compounds in both series, the unsubstituted C5 analogue (MB442) and 4-*tert*-butyl C3 analogue (MB327) had log IC_50_ [M] values of −2.56 and −2.86, respectively, with Hill slopes of −0.60 and −0.89.

As noted, a number of bispyridinium oximes can block nicotinic AChR ion channels and demonstrate recovery in nerve agent-poisoned phrenic nerve hemidiaphragm preparations [16,44]. The degree of recovery ranged from ~10% for HI-6 to recovery approaching ~30% with the 4-*tert*-butyl C3 analogue (MB327), unsubstituted C5 analogue (MB442) and 4-*tert*-butyl C2 analogue (MB583); the symmetrically substituted oxime trimedoxime (TMB-4, Fig 1) being the most effective drug in the previous investigation [16]. The recovery was accomplished at lower concentrations of bispyridinium non-oximes in the current study (10–100 µM) rather than HI-6 or TMB-4 (200 µM) used previously. In the CN21 cell line, HI-6 exhibited a log IC_50_ of −2.76, similar to the most effective bispyridinium non-oximes. These data and the diaphragm recovery suggest the nature of the antagonism, rather than its potency, is an important factor determining efficacy (trimedoxime has not been tested in the CN21 cell line assay) [40].

One of compounds in the present study, MB408 (unsubstituted C3), was also tested in the past [16]. It produced largely similar degrees of recovery in the two studies, although assumptions on the slope of the concentration-response curve must be made, due to differences in the concentrations of the compound used in the two studies. Most of the other studies on substances that cause organophosphorus-intoxicated diaphragm preparations to recover have been carried out with oxime reactivators of AChE [44–48]. After ageing of the OP-AChE addition product, the reactivation from oximes lessens and often recovery diminishes. Many of the substances in the current study seemed to cause a more potent recovery of neuromuscular function in the soman treated diaphragm than that observed in earlier work.

An exception is a study by French *et al.* [44] where greater recovery was observed with some non-oximes compared to that demonstrated here or previously [16]. In the French *et al.* study, test compounds were added 0.5 h after soman intoxication. In the present and earlier study [16], the test compounds were added 1 h after the soman, when ageing of the inhibited enzyme would have completed. Additionally, other chronic effects of the accumulation of ACh in the synapse, such as receptor desensitisation [49,50] produced by mechanisms that may include phosphorylation [51] or internalisation [52], would have occurred to a greater extent after 1 h. Depending on the severity of these effects, the amplitude of the endplate potential would reduce and perhaps drop below the threshold for triggering an action potential in some muscle fibres. The compounds are unlikely to reverse the desensitisation: the longer the preparation stands after soman poisoning, the greater the probability of compounds acting on the nAChR becoming less effective or worsening the situation. Further work on the effect of the bispyridinium non-oximes immediately after soman poisoning would be required to examine this. If the compounds reduce the rate of desensitisation, they may produce recovery more effectively in soman-poisoned diaphragm.

The recovery of neuromuscular function by the test compounds was reversible, i.e., the diaphragm response returned to a state similar to that of the vehicle control group following washout of those compounds that caused recovery. This effect was also possible with most of the compounds that initially produced recovery and then inhibited the response at higher concentrations. These results suggest the mechanism by which the compounds produce the recovery is readily reversible and consistent with the possibility that the inhibition may arise from an overwhelming effect of the same action that produces recovery. However, the test compounds that did not produce recovery and abolished the response completely, the 4-*tert*-butyl C10 and unsubstituted C9 and C10 analogues, were not as easily washed out, and in this case, the function returned much more slowly. The mechanism underpinning this action may differ from the one underlying the recoveries seen with the other test compounds.

These results and those for the nicotinic response in CN21 cells suggest inhibition of diaphragm function is due to antagonism of the nicotinic response to such a degree that transmission failure occurs. In the unsubstituted series, C5 (MB442) produced recovery of diaphragm function, whereas C7 (MB777) and C10 (MB779) were unable to do this over a broad concentration range. If recovery and inhibition are solely due to nicotinic antagonism, then the nature of this antagonism may differ between the C5 and C7/C10 compounds.

In addition to inhibiting nAChR-induced Ca^2 +^ responses, the test compounds inhibited hAChE *in vitro*. This effect, paralleling that observed for inhibition of the nAChR, increased as the linker lengthened, hinting that the positions of the quaternary nitrogen atoms and size and shape of the molecule are important for determining the extent of this action. It is unsurprising that as the compounds match more closely the ACh-binding site on the nicotinic receptor, they also bind more effectively to hAChE: both sites bind ACh and have appreciable structural homology [53].

The anticholinesterase activity of some of the members of the unsubstituted series, but not for hAChE, has been reported [35,36,54,55]. These studies suggest the same leap in AChE inhibition passing from C5 to C6, and increasing to C10. Measurements of hAChE inhibition constants for the 4-*tert-*butyl series [32] showed the same trend: modest inhibition potency for C1–C3, moderate potency for C4 and C5, and a step change in potency for C6 to C10, increasing to a maximum at C10 [56].

Toxicity data are available for various bispyridinium dibromides. They reveal that the LD_50_ in mice diminishes as the linker extends (Table 2). The LD_50_ values for the analogues with the shorter linkers compare favourably to those for several oximes used in nerve agent treatments: HI-6, P2S, 2-PAM, and obidoxime. The present study shows that as the linker of the bispyridinium non-oximes lengthens, the compounds more potently inhibit the nAChR-mediated calcium response and hAChE. This increasing potency of inhibition of the nicotinic response and AChE activity on extending the linkers could account for the increased toxicity moving from C1 to C10.

Previously we showed that MB327 dimethanesulfonate administered with hyoscine and physostigmine improved survival of guinea-pigs pre-treated with 5 × LD_50_ s.c. of soman sarin or tabun after 6 h [9,21]. MB327 dimethanesulfonate, in combination with atropine and avizafone, also provided a degree of protection equivalent to that of HI-6 against soman challenge when survival was assessed after 24 h [25]. Whilst for HI-6 this protection is likely to be due to some reactivation of unaged soman-inhibited AChE, this would not be the case for MB327, which lacks an oxime group. MB327 was also shown to clear rapidly from the circulation (plasma) with a half-life (t_½_) of ~22 min [57]. Given its mechanism of action, it would be expected that the efficacy of MB327 would be reduced as it clears from the circulation and the tissues; protection would be expected to be greater at earlier time points. Indeed, this has been shown clearly with MB327 dimethanesulfonate providing significantly greater protection compared to HI-6 when a 6 h endpoint is used for LD_50_ determinations rather than a 24 h endpoint [57]. Here, we demonstrate that the unsubstituted C5 analogue (MB442) can also improve the survival of guinea-pigs intoxicated by soman when co-administered with hyoscine and physostigmine providing complete protection against 5 × LD_50_ at 6 h. MB442 also enhanced the survival of soman-challenged guinea-pigs when given with atropine and avizafone, but a more modest protection was observed at the 24 h endpoint used in these studies. It is likely that if MB442 exhibits similar rapid clearance then a shorter experimental endpoint would reveal significantly improved protection. It should be noted that in all the efficacy studies described, treatments were administered on one occasion shortly after nerve agent administration. Repeated administration of a bispyridinium (MB327 or MB442) might therefore be predicted to provide improved protection over a longer duration. This has not yet been tested experimentally. The highest dose of MB442 used in the efficacy studies described here (28.78 mg·kg^−1^) would not be considered for future studies due to adverse effects.

These studies show that the experiments used to select the non-oximes are helpful and that the 4-*tert*-butyl group of MB327 assists, but is not vital, to therapeutic activity. The results complement those in mice, where MB327 [34] and the unsubstituted analogues MB408 (C3), MB444 (C4) and MB442 (C5) [58–61] were tested as part of the standard antidotal treatment (atropine and an oxime) against sarin, soman, tabun and cyclosarin. Those experiments and the ones reported here demonstrate the potential of these compounds as adjuncts to the usual nerve agent therapies in two animal species, and the value of a 6 h end-point for determining their effectiveness [57].

That certain bispyridinium oximes such as HI-6 and trimedoxime exhibit both AChE reactivation and nAChR-directed effects might suggest development of additional treatment adjuncts is unnecessary. However, optimisation of both activities in an effective and safe therapeutic compound is likely to be difficult with two unconnected mechanisms of action to consider. For example, in combination with physostigmine and hyoscine the antinicotinic action of HI-6 is insufficient to provide protection against tabun in guinea pigs, whereas an equimolar dose of MB327 dimethanesulfonate exhibited good protection [21]. Additionally, in soman-challenged guinea pigs MB327 could provide protection against much higher challenge doses of soman than could HI-6 [57]. Trimedoxime has comparable actions in the diaphragm assay to the bispyridinium non-oximes, however, it is considered to be one of the most toxic oximes of those that have been tested in humans and therefore has seen limited development and clinical use [7]. These considerations underpin the approach of identifying and using antinicotinic therapeutic compounds to complement standard oxime-based therapeutic approaches. Providing an antinicotinic treatment adjunct is particularly valuable if the agent-inhibited AChE is insensitive to oxime-based reactivation.

## Conclusions

This study confirms the capacity of certain bispyridinium non-oximes to inhibit muscle nAChRs and presents structure-activity relationships. The inhibition of nAChRs and hAChE increases as the linker between the pyridinium rings lengthens. Prior studies showed that the 4-*tert*-butyl C3 analogue, MB327 dimethanesulfonate, protected guinea-pigs from nerve-agent intoxication [20,21]. Here we show that the unsubstituted C5 analogue (MB442) can also protect guinea-pigs intoxicated by soman.

The toxicity of the non-oximes increases with linker length. Data are available only for some of these substances in mice and toxicities for the short-linker analogues compare with those of several oximes used commonly to treat nerve agent intoxication. Overall, these results indicate that non-oximes are promising candidates for treating the nicotinic effects of poisoning from organophosphorus anticholinesterases, and that the pharmacological experiments reported can help optimise molecular structures researched for antidotal use. Work is ongoing to identify the precise mechanism(s) of binding of the efficacious non-oximes to the nAChR [62–67] that underpin their therapeutic properties, especially in human muscle [68]. The structure-activity data presented should help accelerate the drug discovery efforts in this direction. Relatively few anti-nicotinic compounds have been assessed for treatment of nerve agent poisoning due to their toxicity, which typically results from overt neuromuscular block [13]. Non-competitive bispyridiniums provide potentially the best approach yet to engaging the nAChR as a therapeutic target.
